# Low and heterogeneous prevalence of glucose-6-phosphate dehydrogenase deficiency in different settings in Ethiopia using phenotyping and genotyping approaches

**DOI:** 10.1186/s12936-018-2437-8

**Published:** 2018-08-02

**Authors:** Getasew Shitaye, Endalamaw Gadisa, Lynn Grignard, Girma Shumie, Wakweya Chali, Temesgen Menberu, Mulualem Belachew, Getaneh Tegegn, Sagni Challi, Jonathan Curry, Laleta Mahey, Tsegaye Hailu, Hassen Mamo, Menakath Menon, Taye Balcha, Abraham Aseffa, Chris Drakeley, Teun Bousema, Fitsum G. Tadesse

**Affiliations:** 1 Department of Biomedical Sciences, School of Medical Sciences, Bahir Dar University, Bahir Dar, Ethiopia; 2 Armauer Hansen Research Institute (AHRI), Addis Ababa, Ethiopia; 3 Department of Immunology and Infection, London School of Hygiene and Tropical Medicine, London, UK; 4 Department of Biomedical Sciences, School of Medical Sciences, Wolkite University, Wolkite, Ethiopia; 5 Department of Biomedical Sciences, Semera University, Semera, Ethiopia; 6 Genomics Division, LGC Group ltd, Hertfordshire, UK; 7 Department of Microbial, Cellular and Molecular Biology, College of Natural Sciences, Addis Ababa University, Addis Ababa, Ethiopia; 8 Department of Biochemistry, School of Medical Sciences, Addis Ababa University, Addis Ababa, Ethiopia; 9 Radboud Institute for Health Sciences, Radboud University Medical Centre, Nijmegen, The Netherlands; 10 Institute of Biotechnology, Addis Ababa University, PO Box 109, Addis Ababa, Ethiopia

**Keywords:** Radical cure, *P. vivax*, G6PD, 8-Aminoquinoline, Haemolysis

## Abstract

**Background:**

8-Aminoquinolines such as primaquine clear mature *Plasmodium falciparum* gametocytes that are responsible for transmission from human to mosquitoes and bring radical cure in *Plasmodium vivax* by clearing dormant liver stages. Deployment of primaquine is thus of relevance for malaria elimination efforts but challenged by the widespread prevalence of glucose-6-phosphate dehydrogenase deficiency (G6PDd) in endemic countries since primaquine in G6PDd individuals may lead to acute haemolysis. In this study, the prevalence of G6PDd was investigated in different settings in Ethiopia using phenotyping and genotyping approaches.

**Methods:**

Community and school based cross-sectional surveys were conducted from October to December 2016 in four administrative regions (Gambela, Benishangul Gumuz, Oromia, and Amhara) in Ethiopia. Finger prick blood samples were collected for G6PD enzyme activity using the CareStart™ G6PD screening test and genotyping of 36 selected single nucleotide polymorphisms (SNPs) located in the *G6PD* gene and its flanking regions.

**Results:**

Overall, the prevalence of phenotypic G6PDd was 1.4% (22/1609). For the first time in the Ethiopian population, the African variant (A−) was detected in 3.5% (7/199) of the limited set of genotyped samples, which were all phenotypically normal. Interestingly, all of these individuals had a variation at the rs2515904 locus. Strong geographical variation was observed for both phenotypic and genotypic G6PDd; three-quarters of the phenotypically G6PDd individuals were detected in Gambela.

**Conclusion:**

A very low prevalence of G6PDd was detected in the present study populations. The presence of the A− variant alongside other *G6PD* mutants and the patchy distribution of G6PDd indicate that larger studies specifically designed to unravel the distribution of G6PDd at small geographical scale may be needed to tailor malaria elimination efforts in Ethiopia to the local context.

**Electronic supplementary material:**

The online version of this article (10.1186/s12936-018-2437-8) contains supplementary material, which is available to authorized users.

## Background

The substantial reduction in the global burden of malaria in the last 15 years prompted a renewed interest in malaria elimination [1]. Vector control interventions and case management with effective anti-malarials remain the cornerstones in the fight against malaria [2]. Current antimalarial drugs primarily target the blood stage asexual parasites that are uniquely associated with the signs and symptoms of the disease; with incomplete activity against mature *Plasmodium falciparum* gametocytes (sexual stages) that are responsible for human-to-mosquito transmission [3] and the dormant liver stages (hypnozoites) in *Plasmodium vivax* that are responsible for multiple relapses weeks or months after the primary infections have resolved [4]. Transmission reduction for both *P. vivax* and *P. falciparum* [5] and radical cure in *P. vivax* [6] are integral parts of the effort to eliminate malaria. Elimination efforts require drugs that not only alleviate symptoms, but also prevent transmission and provide a radical cure [7].

Primaquine (PQ), an 8-aminoquinoline, is the only licensed drug to date that can clear circulating mature gametocytes and the hypnozoites of *P. vivax* [8, 9]. However, utilization of PQ is hindered by the association of glucose-6-phosphate dehydrogenase deficiency (G6PDd) and PQ-induced haemolysis [10–12]. The human *G6PD* gene, located on the X-chromosome [13], codes for a cytosolic enzyme [14] with key roles to produce the reducing equivalent nicotinamide adenine dinucleotide phosphate (NADPH) that is required to protect cells, including red blood cells (RBCs), from oxidizing agents [15]. The *G6PD* gene has multiple mutant alleles which entail a decrease in enzyme activity; expressing the G6PD deficient (G6PDd) phenotype [16]. In humans, G6PDd is the most common enzyme defect [17] that affects close to 400 million people worldwide [18]. More than 200 different mutations or combinations of mutations that are responsible for gradients of clinical conditions have been identified. The *G6PDd* variants may remain asymptomatic in the absence of triggers but may result in haemolytic anaemia induced by medications, infection, or ingestion of certain foods due to oxidative stress. Any person with RBC G6PD activity < 30% of the normal adjusted male median enzyme activity is considered G6PDd while > 80% is considered normal [19]. Strong regional patterns in the distribution of variants have been reported [20]. The Mediterranean variant (rs5030868) exhibits exceedingly low residual enzyme activity (< 1%) [21] while the Asian variants are more diverse [22] and could cause adverse effects (5–32% residual activity) [23]. The common *G6PD* allelic variants in sub-Saharan Africa are the wild type G6PD*B, G6PD*A+ (a non-deficient variant, A376G), and G6PD*A− (the deficient variant, A376G plus G202A) [16, 17]. G6PD*A− variants with substitutions at G542T, G680T or T968C were also identified [24, 25]. Compared to the Mediterranean (rs5030868) and South East Asian variants, the African variant is generally considered to result in a milder degree of deficiency and less severe haemolysis [20]; although incidents of life-threatening haemolysis have been reported for G6PD A− [26].

Recent estimates indicated that the number of new cases and deaths attributable to malaria in Ethiopia declined by 96.5% and 88.7%, respectively, between 1990 and 2015 [27]. Unlike most of Africa, *P. vivax* is uniquely highly prevalent in the country. Ethiopia contributed towards 10% of the global vivax malaria cases in 2016 [28]. Ethiopia set an ambitious plan of eliminating malaria from selected low-endemic districts by 2020 [29]. The World Health Organization (WHO) recommends single low dose PQ as a *P. falciparum* gametocytocide and a 14-days course for radical cure of *P. vivax* [30]. PQ, which was used in the country for over a quarter of a century before it was removed from treatment regimens for no documented reasons in 1990 [31], is currently reincorporated into the treatment policy of Ethiopia in elimination settings. However, very little evidence is available on the distribution of G6PDd in Ethiopia and the associated mutations. With the move to deploy PQ widely it is timely and important to generate evidence on G6PDd in the Ethiopian population. In this study, G6PDd was investigated in different malaria endemic settings in the Ethiopian population using enzymatic assay and multiple allelic forms of *G6PD* were analysed by genotyping.

## Methods

### Study sites and population

**Fig. 1:**
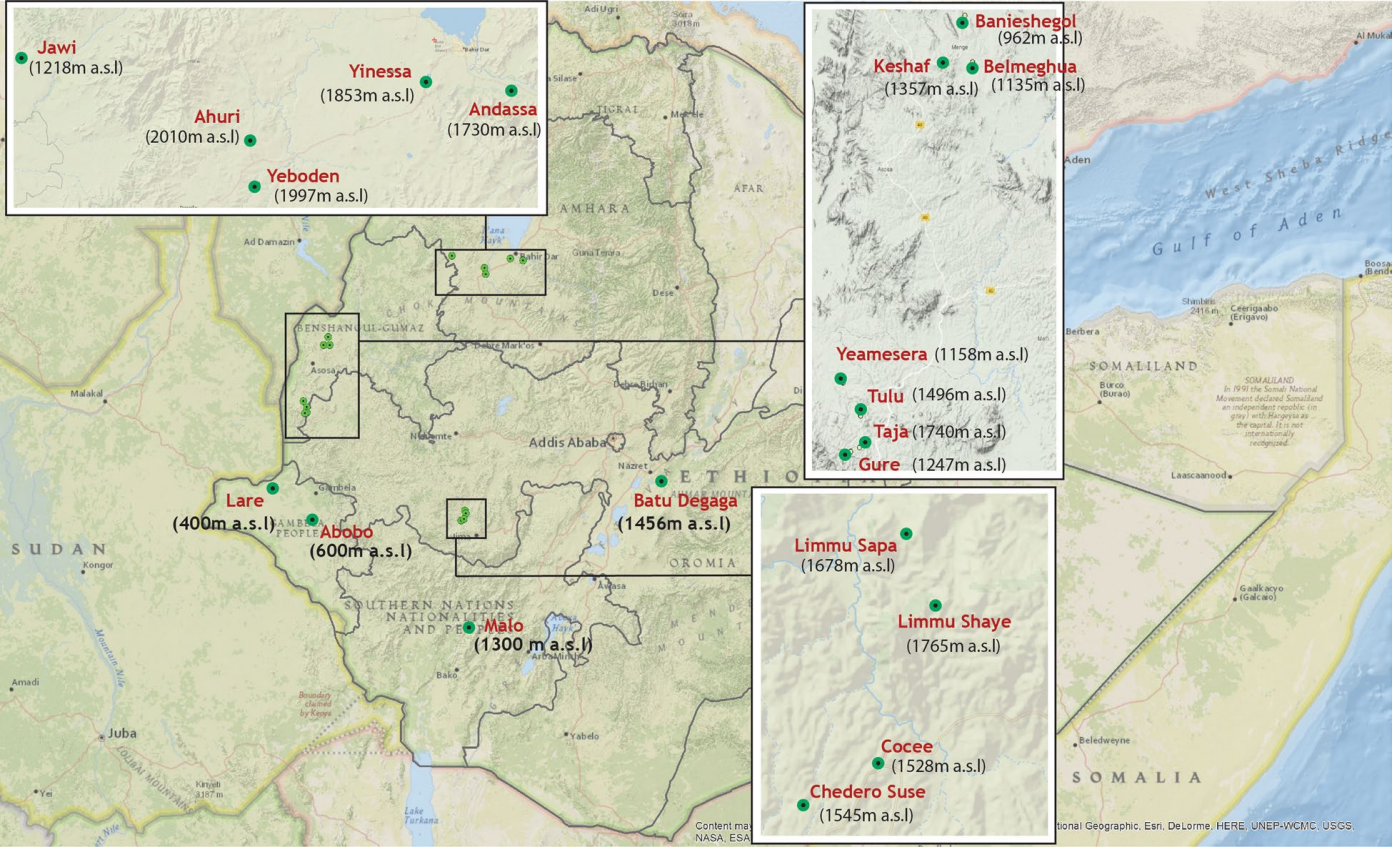
Map of study sites. Community surveys were conducted in Lare and Abobo Woredas of Gambela Regional State, Meng (in Banieshegol, Keshaf and Belmeguha Kebeles) and Maokomo (in Yeamesera, Tulu, Taja and Gure Kebeles) Woredas of Benishangul-Gumuz Regional State, Goma (in Limmu Sapa, Limmu Shaye, Cocee and Chedero Suse Kebeles) and Adama (Batu Degaga Kebele) Woredas of Oromia Regional State. School surveys were conducted in South Achefer (Yinessa and Yeboden Kebeles), Bahir Dar Zuria (Andassa Kebele), and Jawi Woredas in Amhara Regional State. Elevation of each site (meters above sea level, m.a.s.l) is indicated in brackets

Community and school based cross-sectional surveys were conducted from October to December 2016 to investigate G6PDd by phenotyping and genotyping approaches in Ethiopia. Nine Woredas (administrative unit equivalent to district) representing different malaria endemic settings were selected from four Regional States (Gambela, Benishangul Gumuz, Oromia and Amhara Regions). Community based cross-sectional surveys were conducted in Abobo and Lare Woredas of Gambela, Meng and Mao-komo Woredas of Benishangul Gumuz, Gomma and Adama Woredas of Oromia Regional States, Fig. 1. Community mobilization was conducted prior to the study. Residents who were willing to participate in the study were requested to come to the study clinic the next day to donate finger prick blood samples. School based surveys were conducted in Amhara Regional State in Jawi (Jawi general primary school), North Achefer (Ahuri primary school), and Bahir Dar Zuria (Andassa primary school) Woredas, Fig. 1. School based surveys were conducted to estimate parasite prevalence [32] and performed as routinely done using methods developed by Brooker and colleagues to obtain an age-balanced set of samples [33].

### Blood sample collection and G6PD phenotypic screening

Socio-demographic data were recorded using a pre-tested questionnaire and individuals were requested to donate a finger prick blood sample (~ 300 µl) that was used for the assessment of activity of the G6PD enzyme using the CareStart™ G6PDd screening test (Access Bio, Inc., New Jersey, USA). The remaining blood was used to prepare dried blood spots (DBS) on WhatMann 3MM (Whatman, Maidstone, UK) filter papers [32]. DBS samples were air dried, individually packed in a cardboard that had the study code and stored in zip-locked plastic bags containing self-indicating silica gel desiccant beads (Geejay Chemicals Ltd) and transported to the laboratory at AHRI and stored at − 20 °C freezer until processed.

### DNA extraction and single nucleotide polymorphism (SNP) genotyping

Genomic DNA was extracted using saponin-chelex dual extraction procedure as described previously [34] from a 6 mm diameter punch that was eluted in 150 µl of a 6% Chelex in DNase/RNase free water solution and stored at − 20 °C until further use. Thirty-six SNPs located in the *G6PD* gene and its flanking regions were selected from those identified in the literature and on public databases based on criteria described earlier [35–37]. Samples that were found deficient with the enzymatic assay (22) and a subset of 211 G6PD phenotypically normal samples, randomly selected from all study sites, were genotyped using Kompetitive Allele Specific PCR (KASP™) (LGC Genomics, UK). For the 36 putative SNP sequences identified, KASP assays were designed, comprising two allele-specific forward primers, and one common reverse primer (Additional file 1). Primer designs were using sequence derived from NCBI dbSNP and each sequence screened for potential surrounding polymorphism using population information held [37]. Alternative primers were designed if another surrounding polymorphism had an alternative allele frequency greater than 1% (Additional file 2). If was not possible to avoid, then ambiguities were incorporated into the primer in order to minimize any potential impact on assay performance. Genotyping reactions were performed in duplicate using the IntelliQube (LGC, Douglas Scientific, USA) to complete all sample DNA arraying, reagent dispensing, plate sealing and fluorescence detection steps for SNP genotyping as described in detail before [37].

### Data analysis

Questionnaire data was checked for completeness and double-entered along with all laboratory results in RedCap (Vanderbilt University, USA) [38]. Statistical analysis was conducted using STATA 13 (StataCorp, TX, USA) and Graph Pad Prism 5.0 (Graph Pad Software Inc., CA, USA). Computation of Linkage Disequilibrium (LD) and haplotype analysis was done using Haploview version 4.2 Software as stated elsewhere [39]. As a quality control, SNPs with greater than 10% missing genotype calls and more than 2% of male genotype calls that were (falsely) called heterozygous were excluded from analysis including individuals with greater than 20% of the SNP data missing (Additional files 1 and 3). Monomorphic SNPs and those with less than 1% frequency were also excluded from LD and haplotype analysis. No sample size calculations were performed prior to data collection; 95% confidence intervals (CI) were presented for all prevalence estimates.

## Results

A total of 1609 individuals with a median age of 14 years (interquartile range [IQR]: 10–30); of whom 46.3% (745/1609) were females, participated in this study. The overall phenotypic G6PDd detected by the CareStart™ G6PDd screening test was 1.4% (95% confidence interval [CI] 1.0–2.1), with considerable variation between study sites. Of the 22 G6PDd individuals 16 were from Gambela Regional State: 6.6% (95% CI 3.5–11.0) of participants from Abobo and 1.5% (95% CI 0.3–4.4) of participants from Lare Woredas (Table 1). Of the phenotypically deficient individuals 9 were females.

**Table 1:** Characteristics of study participants

Region	Malaria prevalence^a^, %	Woreda	Age in years, median (IQR)	Female sex, % (n/N)	Phenotypic G6PD deficiency, proportion deficient (n/N)		SNP frequency among G6PD normal individuals, proportion deficient (n/N)					
							A376G			G202A		
					Male	Female	Male hemizygotes	Female heterozygotes	Female homozygotes	Male hemizygotes	Female heterozygotes	Female homozygotes
Gambela	18.4	Abobo	13 (8–25)	46.5 (92/198)	7.5 (8/106)	5.4 (5/92)	0 (0/5)	37.5 (3/8)	0 (0/8)	0 (0/5)	11.1 (1/9)	0 (0/9)
		Lare	14 (9–30)	46.0 (91/198)	1.9 (2/107)	1.1 (1/91)	8.3 (1/12)	0 (0/8)	12.5 (1/8)	0 (/12)	0 (0/8)	0 (0/8)
Benishangul Gumuz	10.4	Meng	11 (6–30)	46.3 (93/201)	1.9 (2/108)	0 (0/93)	18.2 (2/11)	33.3 (2/6)	16.7 (1/6)	0 (0/11)	17.7 (1/6)	0 (0/6)
		Mao-Komo	16 (10–35)	42.4 (84/198)	0 (0/114)	0 (0/84)	0 (0/11)	40.0 (4/10)	0 (0/10)	0 (0/11)	0 (0/10)	0 (0/10)
Amhara	1.1	Bahir Dar Zuria	13 (12–14)	46.9 (52/111)	0 (0/59)	0 (0/52)	0 (0/8)	11.1 (1/9)	22.2 (2/9)	0 (0/7)	0 (0/9)	0 (0/9)
		Achefer	13 (12–14)	52.9 (55/104)	0 (0/49)	0 (0/55)	0 (0/6)	16.7 (2/12)	0 (0/12)	0 (0/6)	0 (0/12)	0 (0/12)
		Jawi	13 (12–14)	50.5 (55/109)	0 (0/54)	0 (0/55)	0 (0/10)	12.5 (1/8)	12.5 (1/8)	0 (0/10)	0 (0/8)	0 (0/8)
Oromia	0.7	Adama	13 (8.5–31.5)	23.8 (20/84)	0 (0/64)	0 (0/20)	17.9 (5/28)	0 (0/9)	11.1 (1/9)	14.3 (4/28)	0 (0/9)	0 (0/9)
		Goma	25 (10–40)	50.8 (203/400)	0.5 (1/197)	1.5 (3/203)	5.0 (1/20)	29.4 (5/17)	5.9 (1/17)	0 (0/20)	5.9 (1/17)	0 (0/17)
Total	0.1	–	14 (10–30)	46.3 (745/1609)	1.5 (13/864)	1.2 (9/745)	8.1 (9/111)	20.7 (18/87)	8.1 (7/87)	3.6 (4/111)	3.4 (3/88)	0 (0/88)

*SNP* single nucleotide polymorphism

^a^Data was obtained from the 2015 National Malaria Indicator Survey (MIS 2015). Results indicate RDT positivity among all age groups screened during the study period. SNP data for A376G and G202A (at the right end of the table) is given for the phenotypically normal individuals only. IQR, 25th–75th percentile

A total of 14 SNPs that did not meet the quality control were excluded from further analysis (Additional files 1 and 3): 1 SNP with > 10% missing genotype calls (rs35228794) and 13 SNPs with more than 2% of male genotype calls that were (falsely) called heterozygous (rs766420, rs915941, rs915942, rs1894260, rs2515906, rs1050757, rs2230037, b36_153426256, rs4898389, rs5986877, rs7879049, rs7053878, rs2004651). Additionally, 28 individuals with more than 20% missing calls were also excluded. Out of the 22 high quality *G6PD* SNPs, 6 were found monomorphic [including the well-known variants in Africa rs76723693 (968T>C); rs5030872 (542A>T); rs137852328 (680) and the Mediterranean variant (rs5030868)]. One SNP (rs12389569) had < 1% minor allele frequency (MAF) (Table 2). Thus, further analyses focused on 15 SNPs that passed the quality control. Four of these SNPs are located in the *G6PD* gene (2 synonymous and 2 missense variants) and the other in flanking regions of the gene; i.e. 3 immediately downstream of *G6PD* gene and the remaining were intergenic (1), intron (5), non-coding exon (2) and 5′ UTR variants (1).

**Table 2:** Single nucleotide polymorphisms detected in the study population

Reference SNP identifier	Chromosome position	SNP frequency by sex, proportion deficient (n/N)			Total SNP frequency, proportion deficient (n/N)		Ancestral: alternate allele	Function
		Male hemizygotes	Female homozygotes	Female heterozygotes	Hemizygous/homozygous	Heterozygous		
rs28470352	153,753,490	8.3 (10/121)	5.8 (5/87)	20.7 (18/87)	5.8 (12/208)	10.1 (21/208)	T:A	Intergenic variant
rs61042368	153,755,336	5.0 (6/120)	1.2 (1/86)	1.2 (1/86)	2.4 (5/206)	1.5 (3/206)	G:A	Downstream gene variant
rs12389569	153,757,734	0 (0/120)	0 (0/85)*	2.4 (2/85)	0 (0/205)	1.0 (2/205)	G:A	Downstream gene variant
b36_153413623	153,760,429	1.7 (2/121)	2.4 (2/85)	3.5 (3/85)	1.9 (4/206)	1.5 (3/206)	G:A	Synonymous variant
rs2230036	153,760,953	5.9 (7/119)	1.2 (1/86)	1.2 (1/86)	2.4 (5/205)	2.0 (4/205)	C:T	Synonymous variant
rs73573478	153,761,564	8.9 (11/123)	2.2 (2/90)	4.4 (4/90)	4.7 (10/213)	3.3 (7/213)	G:A	Non coding exon variant
rs5986990	153,761,628	7.8 (9/115)	5.9 (5/85)	21.2 (18/85)	6.0 (12/200)	10.0 (20/200)	G:A	Non coding exon variant
rs2515905	153,762,075	3.3 (4/120)	1.2 (1/87)	4.6 (4/87)	2.4 (5/207)	1.9 (4/207)	G:A	Intron variant
***rs2515904***	***153,762,771***	***3.3 (4/121)***	***1.2 (1/87)***	***4.6 (4/87)***	***2.4 (5/208)***	***1.9 (4/208)***	***G:C***	***Intron variant***
*rs1050829*	*153,763,492*	*8.3 (10/121)*	*7.9 (7/89)*	*20.2 (18/89)*	*6.7 (14/210)*	*10.0 (21/210)*	*A:G*	*Missense variant*
*rs1050828*	*153,764,217*	*3.3 (4/120)*	*0 (0/90)*	*3.3 (3/90)*	*1.9 (4/210)*	*1.4 (3/210)*	*G:A*	*Missense variant*
rs762515	153,764,528	7.7 (9/117)	5.8 (5/86)	20.0 (17/86)	5.9 (12/203)	9.4 (19/203)	T:C	Intron variant
rs762516	153,764,663	3.3 (4/121)	1.2 (1/87)	4.6 (4/87)	2.4 (5/208)	1.9 (4/208)	C:T	Intron variant
rs113492957	153,773,062	9.2 (11/120)	2.3 (2/86)	3.5 (3/86)	4.9 (10/206)	2.9 (6/206)	C:T	Intron variant
rs111827785	153,775,785	18.2 (22/121)	9.6 (8/83)	24.1 (20/83)	12.8 (26/204)	11.8 (24/204)	T:C	5′ UTR
rs60030796	153,836,171	5.0 (6/119)	4.6 (4/87)	12.6 (11/87)	3.4 (7/206)	6.8 (14/206)	A:G	Downstream gene variant

*SNP* single nucleotide polymorphism

The following SNPs were monomorphic and excluded from further analysis: rs5030869, rs76723693 (968), rs137852328 (680), rs5030868, rs5030872 (542), and rs137852318. *SNP with minor allele frequency < 1% and was omitted from further analysis. Italicized are the well-known rs1050828 (G202A) and rs1050828 (A376G) African polymorphisms. Bolded and italicized (rs2515904) indicates a SNP that was detected in individuals who carried the 376 and 202 variants

Among the common SNPs genotyped, the A376G mutation was detected in 34 individuals (17.1%, 95% CI 12.1–23.0) (Tables 1 and 2); 9 (8.1%, 95% CI 3.8–14.8) hemizygous males, 7 (8.0%, 95% CI 3.3–15.9) homozygous females, and 18 (20.7%, 95% CI 12.7–30.7) heterozygous females. In three male participants, a heterozygous signal was observed and confirmed upon repeating the assay. The G202A mutation was detected in 7 (3.5%, 95% CI 1.4–7.1) individuals of whom 4 (3.6%, 95% CI 1.0–9.0) were hemizygous males and the remaining (3.4%, 95% CI 0.7–9.6) were heterozygous females. All G202A hemizygous males were hemizygous for the A376G mutation and of the G202A heterozygous females 2 were homozygous and 1 was heterozygous for the A376G mutation. The African variant was not detected among the phenotypically deficient individuals, only one male phenotypically deficient individual had hemizygous A376G variant. That same individual was hemizygous for rs28470352, rs5986990, rs762515, rs111827785, and rs60030796 variants. The remaining phenotypically deficient individuals had wild type variants for all the SNPs analyzed in this study. Taken together, all individuals that carried the G202A variant also carried the A376G mutations and the A− African variant could not explain the phenotypic variation observed in the present study. All individuals with the A− variant (male hemizygotes and female heterozygotes) were found carrying a variation at the rs2515904 locus (4.6%, 95% CI 2.1–8.5). Like the African variant, the variation at the rs2515904 locus was detected only in phenotypically normal individuals, Table 2. In a similar fashion to the geographic distribution of the phenotypic G6PDd, 4/7 (57.1%) of the A− variants were detected from Adama; they were detected from two families each and two of them were brothers (Table 1). The G202A variant showed high geographic clustering, whereas the A376G mutation was uniformly distributed across all study sites.

**Fig. 2:**
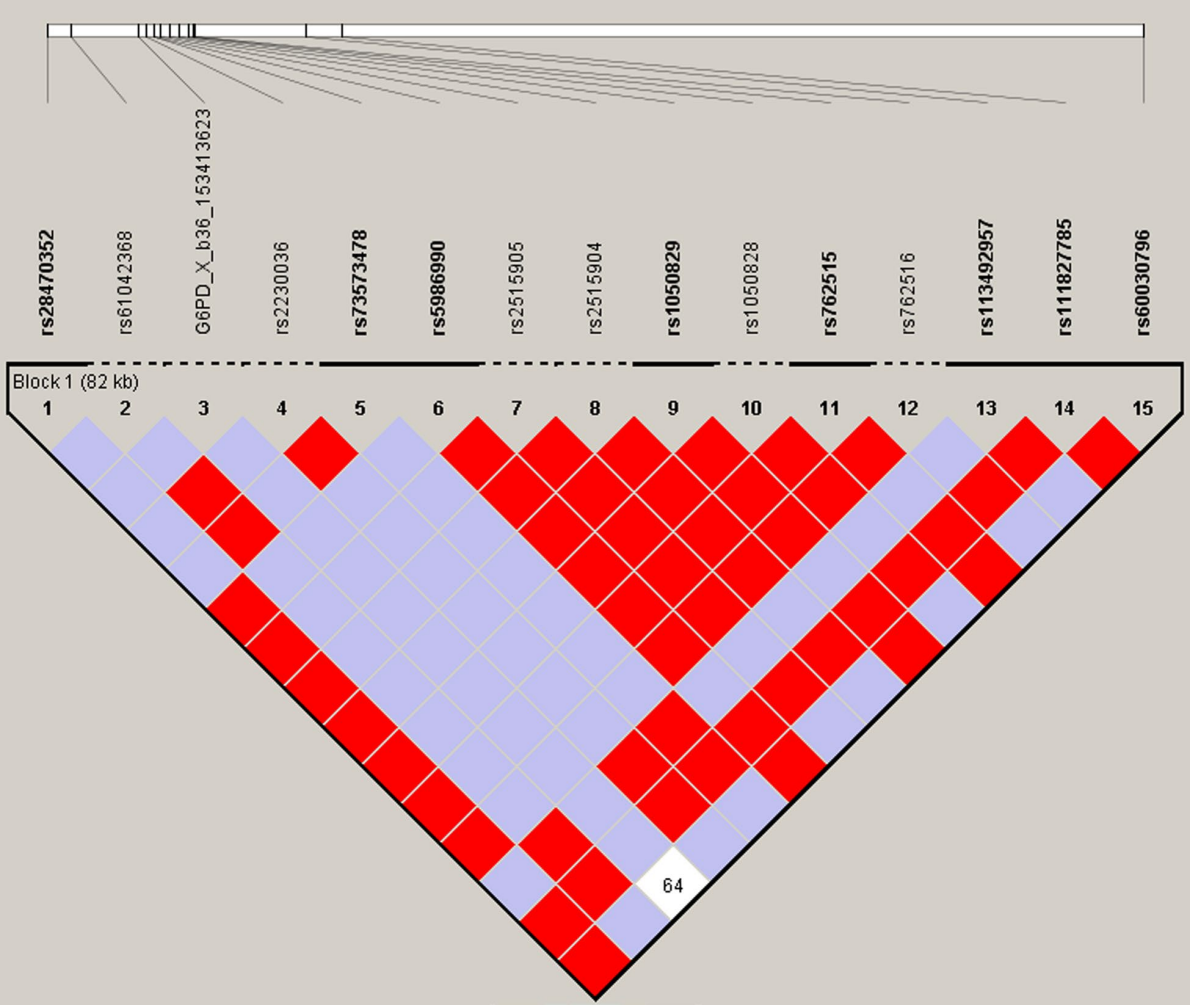
Linkage disequilibrium (LD) plot estimated by Haploview. The numbers in the diamonds are D′ values, with red and no numbers representing a D′ value of 1 for the corresponding pair of variants. Strong LD is indicated by dark gray, whereas light gray and white indicate uninformative and low confidence values, respectively. LD blocks were created with the default algorithm in Haploview that creates 95% confidence bounds on D′ considered to be in strong LD, where 95% of the comparisons made are informative

Linkage disequilibrium (LD) plot could be constructed only for the whole population. It was not stratified based on gender, ethnicity and study setting due to small samples genotyped. One LD block was constructed that consisted of rs28470352, rs73573478, rs5986990, rs1050829 (376), rs762515, rs113492957, rs111827785, and rs60030796 (Fig. 2). Pairwise LD was high in this study (combined gender result was median 1.00; range 0.64–1.00). Generated haplotypes were listed in Table 3.

**Table 3:** Haplotype frequencies in phenotypically normal participants

Haplotypes	Haplotype frequencies
TGG**A**TCTA	0.812
AGA**G**CCCG	0.076
TAG**A**TTCA	0.056
AGA**G**CCCA	0.045
TGG**A**TCCA	0.011

SNPs refer to the following corresponding to Table 2: rs28470352, rs73573478, rs5986990, rs1050829 (376), rs762515, rs113492957, rs111827785, rs60030796. 376 are bolded

## Discussion

A very low frequency (1.4%, 22/1609) of phenotypically evident G6PDd was detected in this study. For the first time in published literature, the African variant (A−) was detected in an Ethiopian population. Of the genotyped samples 3.5% (7/199) were A− variants (4 hemizygous males and 3 homozygous females) and consisted of both the G202A and the A376G mutations. Three quarters of the phenotypically deficient individuals were from one Region, Gambela. None of the phenotypically deficient individuals were found to have the A− variant. Uniquely, all individuals with the A− variant were found carrying a variation at the rs2515904 locus that was previously found in perfect LD with the A− variant and was reported protective against clinical malaria [40–42].

The high prevalence of G6PDd detected by the phenotypic assay in Gambela Region is in line with a recent finding of 7.3% phenotypic deficiency in the same region [43] and with an earlier finding (of 4.0%) among tribes in Eritrea [44]. According to the 2015 National Malaria Indicator Survey, Gambela Region reported the highest prevalence of malaria in Ethiopia (18.4% by RDT compared to 0.1% country wide prevalence) [45] and it is one of the areas in the country with historically stable malaria transmission [46–48]. This might be attributable to its location within the humid tropical region of the country (< 500 masl), settlement and land-use changes [46], and periodic flooding [49]. The geographic overlap of the high malaria and G6PDd observed in this study is supportive of the long-standing claim that this mutation was naturally selected by malaria [50].

Phenotypically evident deficiencies detected in G6PDd individuals often have matched genetic background (often of point mutation or haplotypes). A to G substitution mutation at the rs1050829 locus (also known as A376G, exon 5) is associated with reduced production of G6PD enzyme that causes a milder variant, *G6PD* A+, resulting in 80% residual enzyme activity compared to normal values [51]. The *G6PD* deficient genotype A− is typically defined by the possession of both the rs1050829 (A376G) allele and G to A substitution at the rs1050828 locus (also known as G202A, exon 4). Type A− mutations cause on average 12% residual G6PD activity [51]. Type A− is predominantly found in people who have African ancestry (and referred to as the African variant). In line with the hypothesis that the mutation leading to the African variant (G202A) might have arisen on a chromosome that carried the silent mutation (A376G), all G202A mutations in the present study were detected in individuals carrying the A376G mutation. Most of the phenotypically G6PD deficient individuals in the present study had no SNPs detected in the *G6PD* gene. There was one phenotypically deficient individual who had the A376G, together with variations at rs28470352, rs5986990, rs762515, rs111827785, and rs60030796 loci, but not the G202A. All other SNPs tested in this study were monomorphic for phenotypically deficient individuals, which might be explained by *G6PD* mutations in the Ethiopian population that were not tested in the present study [52, 53]. A relevant shortcoming of the current study was that selected populations of G6PDd and G6PD normal individuals were included in the genotyping assays; the unavailability of an unbiased population makes it challenging to derive meaningful allele frequencies. An additional major limitation was that the sample size was too small to estimate the frequencies of phenotypic G6PDd and mutations in the *G6PD* gene, as well as their associations with RDT performance with precision. The very small sample size, especially with the genotyping analysis, could also have contributed to the observed geographic clustering. The detection of the African variant of G6PDd in the present study and the predominant geographic pattern of patchy distribution of G6PDd underline the need for larger studies to have complete picture of the nationwide distribution of the deficiency to help build a resilient programmatic approach in the move towards eliminating malaria in Ethiopia.

With the limited number of genotyped samples from different study sites, 3.5% (7/199) of genotyped samples were of the A− variant. This contrasts with, previous studies conducted in different settings in Southwest Ethiopia, South Omo [34] and Jimma [52] that did not detect the African A− variant among their Ethiopian populations. One of the unique differences may lie in the study population; the previous studies were all done on clinical patients [52]; possibly missing variants that infer protection from clinical malaria. The other possible explanation is that these studies only included single settings, possibly missing the strong regional patterns in the distribution of *G6PD* variants [20] and differences in ethnic background [43, 54, 55] as demonstrated in the present study. Apart from this the results from this study on the A376G variants (17.1%, 34/199) are in line with a recent finding from Jimma (23.3%) [52]. The Mediterranean variant (rs5030868, C563T) was not detected in any of these studies, including the present study [34, 52]. Allelic heterogeneity, the phenomenon in which different mutations at the same locus cause the same phenotype, might have contributed to the discrepancy reported in previous studies as most of them relied on restriction digestion [34]. Allelic variations can arise as a result of natural selection processes, exogenous mutagens, genetic drift, or genetic migration.

Mutations of *G6PD* can cause varying degrees of G6PDd and thus severity of haemolysis may vary. The patterns of oxidative haemolysis are well characterized in hemizygous males and homozygous females; whereas, heterozygous females may exhibit symptoms to varying degrees due to random inactivation of X-chromosomes [56]. The expression of G6PDd differs markedly among female heterozygotes as their RBC populations are variable mosaics of deficient and normal cells [57] and can potentially be misclassified by currently available phenotypic diagnostic approaches. Although rapid tests that can be used at point-of-care were recently introduced for the diagnosis of G6PDd [58, 59], they may fail to accurately identify individuals with intermediate deficiency (30–80% normal enzymatic activity, mainly heterozygote females) [19]. Heterozygous females who test normal or not deficient in qualitative G6PD screening tests can still experience substantial hemolysis [56]. A quantitative technique is thus required to detect the intermediate levels of G6PD activity but this is not yet available as a point-of-care test and will have cost and logistics implications to overcome when developed and scaled-up [60, 61].

A single low dose PQ as a gametocytocide in *P. falciparum* malaria and a 14-day course for radical cure in *P. vivax* is recommended by the WHO and this is being incorporated into country treatment policies. Most *P. vivax* malaria-endemic countries recommend PQ treatment without prior G6PD testing [62]. The WHO malaria treatment guidelines address the 14-day course for preventing relapse with caution, including a statement that good practice requires that the G6PD status of patients should be ascertained prior to administration of PQ [19]. On top of costs associated with deployment of G6PDd diagnostic tests, the largest challenge is the lack of standardized point-of-care diagnostic tests to-date to mitigate the risk of haemolysis associated with drugs like PQ. Alternative strategies need to be explored in the Ethiopian settings where the frequency of G6PDd is very low and varies substantially between sites; blanket deployment of diagnostic tests might not be cost-effective. Strategies such as directly observed treatment (DOT), close medical supervision for potential PQ-induced haemolysis, as in tuberculosis care [63], using its extensive health extension workers program (with counseling on how to recognize symptoms and signs of haemolytic anaemia) or approaches tailored to local context considering the varying geographic distribution of the deficiency could be plausible options.

## Conclusion

Prevalence of G6PDd is generally very low and varies substantially between settings in Ethiopia. The country could potentially benefit from tailored deployment of 8-aminoquinolines like PQ. This study supports the evidence that the incorporation of PQ into national treatment guidelines for *P. falciparum* malaria would likely pose little risk to the population due to the very low genotypic and phenotypic prevalence of G6PDd. Despite the low prevalence, alternative strategies including DOT or at least better screening for G6PDd, particularly among females, who are likely to be phenotypically normal by Carestart G6PD screening test, but may experience substantial haemolysis if they were genotypically female heterozygous, need to be considered to prevent *P. vivax* relapse.

## Additional files

**Additional file 1.** Flow chart for genotyping and data analysis. Indicated in a) is the schematic presentation of the quality check for data analysis and in b) is the genotyping procedure.

**Additional file 2.** KASP Primer sequences.

**Additional file 3.** Genotyping overview.
