# Supplementary figures and images for: Low and heterogeneous prevalence of glucose-6-phosphate dehydrogenase deficiency in different settings in Ethiopia using phenotyping and genotyping approaches
Source: Malar J. 2018 Aug 2;17:281. doi: 10.1186/s12936-018-2437-8 (PMC6071387; doi:10.1186/s12936-018-2437-8)

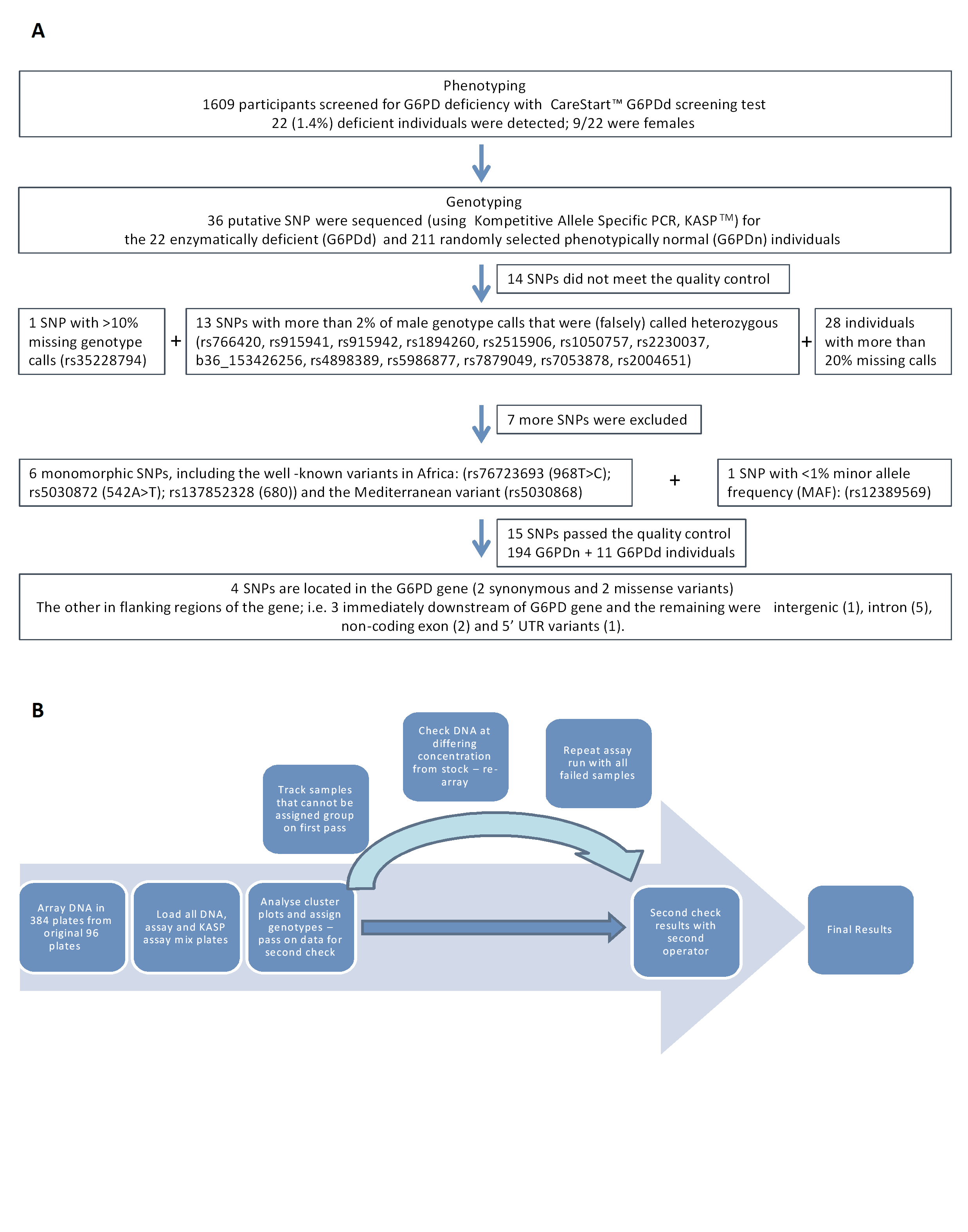

Supplement: Supplementary file 1 — Additional file 1. Flow chart for genotyping and data analysis. Indicated in a) is the schematic presentation of the quality check for data analysis and in b) is the genotyping procedure. [file 12936_2018_2437_MOESM1_ESM.tif]
